# Serum cytokine profile and clinicopathological findings in oral lichen planus, oral lichenoid lesions and stomatitis

**DOI:** 10.1002/cre2.91

**Published:** 2017-11-07

**Authors:** Kristine Roen Larsen, Jeanne Duus Johansen, Jesper Reibel, Claus Zachariae, Anne Marie Lynge Pedersen

**Affiliations:** ^1^ Section for Oral Pathology and Oral Medicine, Department of Odontology, Faculty of Health and Medical Sciences University of Copenhagen Denmark; ^2^ National Allergy Research Centre, Department of Dermatology and Allergy Gentofte University Hospital Denmark; ^3^ Department of Dermatology and Allergy Gentofte University Hospital Denmark

**Keywords:** Generalized stomatitis, oral lichenoid lesions, oral lichen planus, serum cytokines

## Abstract

The objective of this study was to examine if clinical and histopathological variables in patients with oral lichen planus (OLP), oral lichenoid lesions (OLL), and generalized stomatitis display different cytokine profiles and if concomitant contact allergy influences this profile. Forty‐nine patients and 29 healthy age‐ and gender‐matched subjects were included. Demographic and clinical data immunohistochemical findings in mucosal specimens, results of contact allergy testing, and serum levels of tumor necrosis factor‐α, interferon‐γ, interleukin (IL)‐6, IL‐10, IL‐12p40, and IL‐12p70 were analyzed and compared between groups. Nineteen patients had OLP, primarily with ulcerative lesions on the buccal mucosa, 19 patients had OLL, and 11 patients had generalized stomatitis. All patients had oral symptoms, mainly stinging and burning. Nineteen patients and 10 healthy subjects had contact allergies, primarily to fragrance ingredients. Patient groups did not differ with regard to oral symptoms, clinical pattern of the lesions, or contact allergy. Serum cytokine levels did not differ between the different patient groups and were not related to histopathological findings. The patients had higher levels of IL‐6 than the healthy subjects. Interferon‐γ, IL‐12p40, and IL‐12p70 were below detection limit. Our findings indicate that OLP, OLL, and generalized stomatitis cannot be discriminated by means of the selected serum cytokines, and that the presence of concomitant contact allergy does not influence the cytokine expression.

## 1 INTRODUCTION

Oral lichen planus (OLP) is a chronic, inflammatory, and immune‐mediated oral‐mucosal disease affecting 0.5% to 2% of the adult population (Axéll & Rundquist, 1987; Bowers, Sexton, & Sugerman, 2000; McCartan & Healy, 2008; Pinto, Khalaf, & Miller, 2015). OLP is about twice as common in women as in men and mostly affects middle aged and older adult (Kovac‐Kovacic & Skaleric, 2000; Scully et al., 1998). The buccal mucosa, the margins of the tongue, and the gingivae are the most commonly involved sites. Clinically, OLP may present as reticular, erythematous, erosive or ulcerative, plaquelike, bullous, and papular lesions of which the latter two are considered uncommon (McCartan & Healy, 2008; Scully & Carrozzo, 2008; Thorn, Holmstrup, Rindum, & Pindborg, 1988). The etiology and pathogenesis remain unclear, but T‐lymphocytes are believed to be involved. The mechanisms that trigger the T‐lymphocytes to enter the oral epithelium and to form the subepithelial inflammatory infiltrate, and the triggering mechanisms underlying basal keratinocyte apoptosis are still not clarified. It may involve both antigen‐specific and non‐specific mechanisms. The inflammatory infiltrate mainly consists of activated cytotoxic (CD8+)‐T‐lymphocytes, which are assumed to interact with CD4+‐T‐lymphocytes, Langerhans cells, macrophages (CD68+), and basal keratinocytes (Sugerman et al., 2002).

The diagnosis of OLP is based on fulfillment of clinical and histopathological criteria (Kramer, Lucas, Pindborg, & Sobin, 1978; van der Meij & van der Waal, 2003). Lesions that clinically and histopathologically resemble OLP may develop as a reaction to certain systemic medications (Porter & Scully, 2000) or dental materials (Bolewska, Holmstrup, Møller‐Madsen, Kenrad, & Danscher, 1990; Lind, 1988; Scully & Carrozzo, 2008) and are referred to as oral lichenoid lesions (OLL). Oral hygiene products containing aroma substances may also trigger lichenoid contact allergic reactions (Miller, Gould, & Bernstein, 1992; Yiannias, el‐Azhary, Hand, Pakzad, & Rogers, 2000). Generalized stomatitis is characterized as a more diffuse reaction varying from barely visible to a bright red erythematous lesion in addition to erosive and hyperkeratotic lesions occurring anywhere in the oral mucosa (Isaac‐Renton, Li, & Parsons, 2015). Generalized stomatitis may derive from allergic reactions to aroma substances in oral hygiene products as well as metals used in dentistry (Isaac‐Renton et al., 2015).

Cytokines play an important role in both innate and adapted immune responses, and changes in the synthesis of cytokines may initiate immune responses that can lead to development of various inflammatory, immune‐mediated diseases. Changes in the expression of cytokines may reflect the extent of immune dysregulation and various roles of cytokines in the pathogenesis of inflammatory diseases such as OLP (Lu, Zhang, Sun, Du, & Zhou, 2013; Moudgil & Choubey, 2011). A number of studies have shown that the expression pattern of various inflammation‐related cytokines, including tumor necrosis factor (TNF)‐α, interferon (IFN)‐γ, and interleukin (IL‐)‐1, 2, 4, 5, 6, 8, 10, 12, 17, 22, in lesions, saliva, serum, and peripheral blood mononuclear cells (PBMCs) from patients with OLP differs from that seen in healthy control subjects (Dan et al., 2011; de Brito Monteiro et al., 2015; Kaur & Jacobs, 2015; Kondo & Urisu, 2009; Liu et al., 2014; Malekzadeh, Robati, Yousefimanesh, Ghafourian Boroujerdnia, & Nadripour, 2015; Pekiner, Demirel, Borahan, & Ozbayrak, 2012; Piccinni et al., 2014; Simark‐Mattsson et al., 1999; Wang, Zhou, Fu, Wang, & Zhou, 2015; Zhou et al., 2009). TNF‐α is one the most studied cytokines in OLP and is believed to play an important role in the immune regulation of OLP (Pezelj‐Ribaric et al., 2004). Levels of TNF‐α have been found increased in OLP lesions and saliva, decreased in PBMCs, whereas findings regarding the levels in serum are inconsistent (Lu et al., 2013; Ma et al., 2016; Sugerman et al., 2002). INF‐γ is produced by T‐helper Type 1 (Th1) cells, whereas IL‐10 is produced by Th2 cells (Bai et al., 2008; Piccinni et al., 2014; Rhodus, Cheng, & Ondrey, 2007). INF‐γ is assumed to be involved in the activation of CD8+ T lymphocytes and to maintain major histocompatibility class II molecules on keratinocytes (Ghallab, el‐Wakeel, & Shaker, 2010; Sugerman et al., 2002). IL‐20 differentiates naïve T cells into Th1 effector cells, induces production of INF‐γ from natural killer cells and T cells and enhances the cytotoxicity of natural killer cells and CD8+ T‐lymphocytes (Gee, Guzzo, Che Mat, Ma, & Kumar, 2009). IL‐12p40 expression has been found increased in OLP lesions, whereas the levels in serum are unknown (Pan, Cai, & Yu, 2011).

Clinically, and in many cases also histopathologically, it may be difficult to discriminate between OLP, OLL, and generalized stomatitis. However, characterization of the potential differences has important clinical and therapeutic implication, for example, considering the potential malignant transformation of some lesions and the potential involvement of allergy. The purpose of this cross‐sectional study was therefore to clarify whether the profile of selected serum cytokines differ between patients with OLP, OLL, and generalized stomatitis and between patients and healthy control subjects, and whether variations in clinical and histopathological findings as well as the presence of concomitant contact allergy have an impact on the serum cytokine profile.

## 2 MATERIALS AND METHODS

### 2.1 Study participants

This study was approved by the ethics committee of the Capital Region of Denmark (No. H‐3‐2013‐033) and conducted according to the Declaration of Helsinki. Informed consent was obtained from all included participants.

One hundred thirty‐four consecutive patients referred the Clinic for Oral Medicine, Department of Odontology, Faculty of Health and Medical Sciences, University of Copenhagen, due to symptoms and signs of oral mucosal diseases were screened for inclusion in the study. Fifty‐two Caucasian patients were enrolled of whom 49 completed all examinations. The remaining patients were excluded due to other conditions than OLP, OLL, and generalized stomatitis, including suspicion of medication‐induced OLL. None of the patients had severe periodontitis, ongoing infections, systemic autoimmune diseases, or received immunosuppressive therapy. Twenty‐nine healthy age‐ and gender‐matched subjects were recruited via the Danish website for research study subjects, www.forsoegsperson.dk. The exclusion criteria for these subjects were past or current history of systemic and oral diseases as well as intake of medication. Four persons taking antihypertensives were matched to the patients regarding age, gender, and type of antihypertensive drug as it proved difficult to recruit nonmedicated control subjects above the age of 65 years.

An oral smear was taken from all participants before inclusion in order to exclude oral candidiasis as it may mimic other mucosal lesions. Besides, a superimposed candidiasis may masquerade the pattern of OLP lesions and cause the oral symptoms. The smear was stained with Periodic acid‐Schiff and evaluated cytologically for presence of yeast hyphae and spores. Oral candidiasis was present in 11 patients, but in none of the healthy subjects. All of them were treated with nystatin for 4 weeks, before inclusion. However, the antifungal treatment had no impact on their oral symptoms, but a repeated smear showed that the hyphae and spores were eliminated.

All patients had oral symptoms and were diagnosed with OLP, OLL, or generalized stomatitis. Clinically, the patients with OLP and OLL were characterized by various combinations of reticular (white striations), erythematous, ulcerative, and plaquelike (homogenous, slightly elevated white patches) lesions, and some of the lesions being in close proximity of one or more dental restorations. The patients diagnosed with generalized stomatitis were characterized by having a more diffuse, widespread oral mucosal erythema. One examiner (K. R. L.) performed the oral clinical examination, calibrated against an experienced clinical examiner (A. M. L. P.). The localization, size, and color of the oral lesions were registered, and clinical photos were taken.

All participants underwent a mucosal biopsy in order to confirm to the diagnosis and to confirm that the healthy control subjects had normal oral mucosa. The histological features of OLP included a well‐defined, bandlike zone of inflammatory infiltrate, confined to the superficial lamina propria, and liquefactive degeneration of the basal cell layer according to the criteria of van der Meij and van der Waal (2003). None of the patients were treated for their symptoms before or during the study.

All participants underwent patch testing for contact allergy at the Department of Dermatology and Allergy, Gentofte University Hospital. Patch testing to the European baseline series, a toothpaste series, and a dental material series were done according to the European Society of Contact Dermatitis guidelines (Johansen et al., 2015).

All participants were asked standardized questions regarding past and current systemic diseases, including allergies, daily intake of medication, habits regarding alcohol consumption, tobacco smoking, and oral hygiene. Data on smoking habits was used to categorize participants as never smokers, former smokers, and current smokers. Data on alcohol consumption was used to pool the participants in groups of never consuming alcohol, occasionally, or daily consumption of alcohol. They were also being questioned about symptoms of the oral mucosa such as itching, burning and roughness, dysgeusia, and xerostomia (Larsen, Johansen, Reibel, Zachariae, & Pedersen, 2017). Additionally, measurements of unstimulated and paraffin‐chewing‐stimulated whole saliva flow rates and stimulated parotid saliva flow rates were performed (Larsen, Johansen, Reibel, Zachariae, & Pedersen, 2017).

Peripheral blood samples were obtained from all participants. The samples were centrifuged to separate to the serum, and then portioned and stored at −80 °C until further analysis. The determination of the different types of serum cytokines were performed using enzyme‐linked immunosorbent assays for determination of human TNF‐α, INF‐γ, IL‐6, IL‐10, IL‐12p40, and IL‐12p70, following the protocol provided by R&D Systems ((DuoSet® ELISA Development Systems, R&D Systems, UK). The recovery rates varied between 57% and 95%.

### 2.2 Statistical analysis

The spss Version 22 (IBM) was used for statistical analysis. Mann–Whitney *U*‐test was used for comparison of the concentrations of TNF‐α, INF‐γ, IL‐6, IL‐10, IL‐12p40, and IL‐12p70 between the patient groups and patient group and healthy control group. The Spearman rank correlation test was used to explore associations between levels of serum cytokines and clinical and histopathological parameters. Fischer's exact test was used for evaluation of differences in the distribution between the patient groups, including those with or without a concomitant contact allergy and the healthy control group. Associations between variables were analyzed by the Spearman rank order correlation test. Statistical significance was set at a *p* value < .05.

## 3 RESULTS

Nineteen patients were diagnosed with OLP and another 19 patients with OLL. Eleven patients had generalized stomatitis. Table 1 shows the demographic and medical data of the patients with OLP, OLL, and generalized stomatitis. About 85% of the participants were women with an average age of about 61 years (range 31–77 years). The patients with generalized stomatitis were slightly older, that is, 65 years than the OLP and OLL patients. Three patients and four healthy subjects smoked cigarettes on a daily basis (mean 21.5 and 17.3 smoking pack years, respectively). Seven patients and 11 healthy control subjects reported that they had a daily consumption of alcohol. There were no differences between the groups with regard to smoking and alcohol habits.

**Table 1:** Demographic and medical data of the patients with OLP, OLL, and stomatitis

	OLP (*n* = 19)	OLL (*n* = 19)	Stomatitis (*n* = 11)
Age, mean ± SD (range), years	60.2 ± 10.3 (46–73)	59.1 ± 10.3 (31–75)	65.5 ± 9.4 (48–77)
Gender (female : male ratio)	17:2	16:3	10:1
Smokers	1	1	1
No. of medical diseases, median	2	2	2
No. of medications, median	2	2	2
Contact allergy	6	9	4
Active dermatoses, not LP (%)	20.4	21.1	18.2
Xerostomia (varying severity)	7	9	6
Taste disturbances	7	3	4
Other oral symptoms^a^			
Stinging, stabbing, burning	17	13	11
Roughness of the oral mucosa	14	15	3
Abrasion of the oral mucosa	6	14	4
Ulceration of the oral mucosa	6	12	4
Blisters in the oral mucosa	7	4	1
Peeling of the oral epithelium	3	14	2

*Note*. OLP = oral lichen planus; OLL = oral lichenoid lesions; LP = lichen planus.

a Some patients had more than one symptom

In the patient group as a whole, 85.7% reported having one or more medical condition or disease (median 2, range 1–12) of which the most prevalent included recurrent labial herpes infection, hypertension, osteoarthritis, contact dermatitis, and asthma. Furthermore, six patients had pollen allergy, seven had contact allergy to nickel, and five had allergy to fragrance ingredients, whereas none of the healthy subjects reported allergies (Larsen, Johansen, Reibel, Zachariae, Rosing et al., 2017).

In the patient group as a whole, 65.3% reported daily intake of prescribed medication (median 2, range 1–10), mostly antihypertensives. About one third of the patients (*n* = 16) had a daily intake of more than two different types of medication. There were no associations between age, gender, and the number of medical conditions or diseases and the number of medication taken on daily basis.

Patch testing revealed that 19 patients (38.8%) and 10 (34.5%) healthy control subjects had contact allergy, mainly to fragrance mix. The patients, and especially those with OLP and OLL, had significantly more contact allergy to aroma substances in oral hygiene products than the healthy control subjects (*p* = .02 and *p* = .01). Spearmint was the most common allergen in the patient group and cassia oil in the healthy control group (Larsen, Johansen, Reibel, Zachariae, Rosing et al., 2017).

The patients had more complaints of xerostomia and more severe xerostomia than the healthy control subjects (*p* < .001). About 47% of the patients reported symptoms of xerostomia varying from slight to severe symptoms, whereas the healthy subjects reported slight sensation of dry mouth, and mainly related to snoring or mouth breathing. The frequency of xerostomia did not differ between patients with OLP, OLL, and generalized stomatitis and patients with and without a concomitant contact allergy. There were no associations between the presence and severity of xerostomia and age, gender, number of medical conditions or diseases, including allergies, or the number of medications taken on a daily basis. No differences could be found between the patient groups and the healthy control group and those with and without a concomitant contact allergy with regard to whole saliva and parotid saliva flow rates (Larsen, Johansen, Reibel, Zachariae, & Pedersen, 2017; Larsen, Johansen, Reibel, Zachariae, Rosing et al., 2017).

All patients reported various oral symptoms related to their oral lesions (Table 1). The most prevalent symptoms were stinging, stabbing, and burning, and patients often reported more than one symptom. There was no difference between the patient groups with regard to symptoms and the number of symptoms reported.

The clinical and histopathological pattern of the oral lesions observed in the patients is presented in Tables 2 and 3, respectively. The most common site for oral lesions was the buccal mucosa. There was no difference between the patient groups with regard to the site of lesions, but in OLP, the lesions were more or less symmetrical. There were no associations between age, gender, symptoms, site, or type of the oral lesions. However, patients with lesions on the gingivae tended to have more oral symptoms. As only a few of the patients were smokers, we did not find any association to the clinical pattern of the lesion and smoking. In general, discrimination between OLP, OLL, and generalized stomatitis based on the clinical observations was very difficult.

**Table 2:** The clinical pattern of the oral lesions in patients with OLP, OLL, and stomatitis

	OLP (*n* = 19)	OLL (*n* = 19)	Stomatitis (*n* = 11)
Clinical pattern			
Reticular (lacelike network of slightly raised gray‐white lines)	19	19	5
Erosive or ulcerative	1	3	0
Plaquelike	2	2	0
Erythematous or atrophic	16	13	11
Bullous	0	0	0
Papular	0	0	0
Site of lesions^a^			
Buccal mucosa	14	17	6
Gingivae	13	15	5
Tongue	8	5	4
Alveolar buccal sulcus	8	11	2
Palate	3	4	3
Labial mucosa	6	4	8
Floor of the mouth	1	0	1

*Note*. OLP = oral lichen planus; OLL = oral lichenoid lesions.

a Some patients had combinations of lesions and lesions at more than one site.

**Table 3:** Histopathological characteristics of the oral lesions in patients with OLP, OLL, and stomatitis

	OLP (*n* = 19)	OLL (*n* = 19)	Stomatitis (*n* = 11)
Histopathological characteristics			
Hyperortho‐ or hyperparakeratosis	19	19	6
Saw‐tooth rete ridges	8	1	0
Civatte bodies or single cell necrosis	9	6	1 or 0
Band of eosinophilic material	12	9	0
Well‐defined, bandlike zone of cellular infiltration of mainly lymphocytes confined to the superficial lamina propria	19	0	0
Liquefaction degeneration in the basal layer	14	8	1
Epithelial atrophy	17	13	3
Epithelial hyperplasia	2	0	1
Diffuse chronic inflammation	3	11	3

*Note*. OLP = oral lichen planus; OLL = oral lichenoid lesions.

In general, the levels of the serum cytokines displayed large variations (Table 4). Serum levels of IL‐6 was significantly higher in the patients than in the healthy control subjects *(p* = .048), whereas TNF‐α and IL‐10 did not differ. There was no statistical significance in the serum levels of TNF‐α, IL‐6, and IL‐10 between the patients with OLP, OLL, and generalized stomatitis and those with and without concomitant contact allergy. All measured serum levels of IL‐12p40 and INF‐γ were below the limit of detection. Serum IL‐12p70 could only be detected in a single patient.

**Table 4:** Serum levels of IL‐6, IL‐10, IL‐12p40, IL‐12p70, TNF‐α, and INF‐γ measured in pg/ml

	OLP	OLL	Stomatitis	Healthy controls
IL‐6	89.6 ± 300.7	50.7 ± 128.3	49.8 ± 106.2	48.3 ± 128.0
IL‐10	4617.6 ± 1806.2	366.7 ± 876.2	464.1 ± 549.4	763.3 ± 2396.8
IL‐12p40	Below limit of detection	Below limit of detection	Below limit of detection	Below limit of detection
IL‐12p70	One sample	Below limit of detection	Below limit of detection	Below limit of detection
TNF‐α	645.9 ± 1597.4	760.3 ± 1563.8	246 ± 179.6	Below limit of detection
INF‐γ	Below limit of detection	Below limit of detection	Below limit of detection	Below limit of detection

*Note*. All data are given in mean and SD. OLP = oral lichen planus; OLL = oral lichenoid lesions.

## 4 DISCUSSION

The aim of this cross‐sectional study was to determine whether the clinical and histopathological characteristics of OLP, OLL, and stomatitis can be reflected in differences in the profile of serum cytokines, and also whether the presence of concomitant allergy influence the levels of selected circulating cytokines. Findings were compared between the various patient groups and also between the patients and the healthy control subjects. Presently, discrimination between OLP and OLL is difficult based on the clinical features, and in some cases, also with regard to the histopathological features, and there is a need to identify markers that can be helpful in this discrimination.

The results of our study did not demonstrate any significant differences in the serum levels of cytokines tested nor between patients with OLP, OLL, and generalized stomatitis or patients with or without contact allergy. Furthermore, only the serum levels of IL‐6 were significantly higher in the patients as a whole when compared to the healthy control group. We found no associations between the clinical pattern of oral lesions and the histopathological features and age, gender, oral symptoms, and serum cytokines.

Several studies have shown that the cytokine profile in saliva, lesions, and serum of patients with OLP differs from that seen in healthy individuals (Dan et al., 2011; de Brito Monteiro et al., 2015; Kaur & Jacobs, 2015; Kondo & Urisu, 2009; Liu et al., 2014; Lu et al., 2013; Malekzadeh et al., 2015; Pekiner et al., 2012; Piccinni et al., 2014; Simark‐Mattsson et al., 1999; Wang et al., 2015; Zhou et al., 2009). In this study, we found that the serum levels of IL‐6 were higher in the patients than in the healthy control subjects and in accordance with previous findings that also found an association between levels of IL‐6 and the stages of OLP (Abdel‐Haq et al., 2014; Kaur & Jacobs, 2015). In our study, the majority of patients (15 out of 19) had erosive or ulcerative OLP lesions at the time of examination.

In this study, we found no difference in the serum levels of TNF‐α and IL‐10 between the patients groups and between the patients and healthy control subjects. The results of previous studies on TNF‐α are conflicting, although mostly showing elevated levels in patients with OLP compared to healthy controls (Kaur & Jacobs, 2015; Kondo & Urisu, 2009; Lu et al., 2013; Pekiner et al., 2012; Simark‐Mattsson et al., 1999; Zhou et al., 2009). The serum levels of IL‐10 has been found increased in patients with OLP (Dan et al., 2011; Pekiner et al., 2012) but also decreased (Lu et al., 2013). The levels of INF‐γ, IL‐12p40, and IL‐12p70 were below the detection limit. Previous studies have shown inconsistent results regarding serum levels of INF‐γ in patients with OLP, reporting both higher (Hu et al., 2013) and no difference compared to healthy controls (Pekiner et al., 2012). Studies on IL‐12 are limited and have shown conflicting results. Ohno et al. (2011) reported in 2011 higher production of IL‐12 by PBMCs from patients with OLP than in those from healthy control subjects. A higher production of IL‐12 has also been shown in the salivary epithelial cells from patients with OLP compared to those from healthy control subjects (Janardhanam et al., 2012), whereas another study found decreased number of IL‐12‐secreting T cells in peripheral blood of patients with OLP when compared to healthy control subjects (Kalogerakou, Albanidou‐Farmaki, Markopoulos, & Antoniades, 2008).

All patients had oral symptoms and mainly symptoms of stabbing, stinging and burning, and also a high frequency of xerostomia. In addition, the patients reported a high number of medical conditions and intake of medication, which is accordance with previous studies showing that women, and especially above the age of 65 years, have more medical diseases, a higher intake of medications and report more symptoms than men (Smidt, Torpet, Nauntofte, Heegaard, & Pedersen, 2011). OLP is estimated to be twice as common in women as in men, but in this study, the ratio was significantly higher (6:1) (Kovac‐Kovacic & Skaleric, 2000; Scully et al., 1998). The age of the patients is in accordance with findings of previous studies showing that OLP and OLL often occur at the average age of 60 years (Kovac‐Kovacic & Skaleric, 2000; Scully et al., 1998). The age of onset also suggest that gender hormones may be involved in the pathogenesis by making the mucosa more susceptible to oral diseases such as OLP and allergic reactions.

## 5 CONCLUSION

In our study the serum levels of TNF‐α, INF‐γ, IL‐6, IL‐10, IL‐12p40, and IL‐12p70 were not useful in the discrimination between OLP, OLL, and generalized stomatitis and between these conditions with or without contact allergic reactions.

## CONFLICT OF INTEREST

None declared.
